# Impact of climate change on vector- and rodent-borne infectious diseases

**DOI:** 10.25646/11401

**Published:** 2023-06-01

**Authors:** Sandra Beermann, Gerhard Dobler, Mirko Faber, Christina Frank, Birgit Habedank, Peter Hagedorn, Helge Kampen, Carola Kuhn, Teresa Nygren, Jonas Schmidt-Chanasit, Erik Schmolz, Klaus Stark, Rainer G. Ulrich, Sabrina Weiss, Hendrik Wilking

**Affiliations:** 1 Robert Koch Institute, Berlin, Germany; 2 Bundeswehr Institute of Microbiology, Munich, Germany, National Consulting Laboratory for Tick-Borne Encephalitis; 3 Robert Koch Institute, Berlin, Germany Department of Infectious Disease Epidemiology; 4 German Environment Agency, Berlin, Germany, Section IV 1.4 Health Pests and their Control; 5 Robert Koch Institute, Berlin, Germany Centre for Biological Threats and Special Pathogens; 6 Friedrich-Loeffler-Institut, Greifswald - Insel Riems, Germany, Institute of Infectology; 7 Bernhard Nocht Institute for Tropical Medicine, Hamburg, Germany, Arbovirus and Entomology Department; 8 Friedrich-Loeffler-Institut, Greifswald - Insel Riems, Germany, Institute of Novel and Emerging Infectious Diseases; 9 German Center for Infection Research Greifswald - Insel Riems, Germany; 10 Robert Koch Institute, Berlin, Germany Centre for International Health Protection

**Keywords:** CLIMATE CHANGE, RESERVOIR HOSTS, VECTORS, ARBOVIRUSES, ZOONOSES, PUUMALA ORTHOHANTAVIRUS

## Abstract

**Background:**

Endemic and imported vector- and rodent-borne infectious agents can be linked to high morbidity and mortality. Therefore, vector- and rodent-borne human diseases and the effects of climate change are important public health issues.

**Methods:**

For this review, the relevant literature was identified and evaluated according to the thematic aspects and supplemented with an analysis of surveillance data for Germany.

**Results:**

Factors such as increasing temperatures, changing precipitation patterns, and human behaviour may influence the epidemiology of vector- and rodent-borne infectious diseases in Germany.

**Conclusions:**

The effects of climatic changes on the spread of vector- and rodent-borne infectious diseases need to be further studied in detail and considered in the context of climate adaptation measures.

## 1. Introduction

Vector- and rodent-borne human diseases and the impact that climatic changes may have on them are an important public health issue regionally, nationally, and globally. Both native and imported vector- and rodent-borne infectious agents can be linked to high morbidity and mortality and impose significant costs on the health care system. Globalisation and climate change also favour the introduction and spread of new vectors and new vector-borne infectious agents, which are accompanied by an expansion of the infectious agent spectrum in Germany.

**Figure 1:**
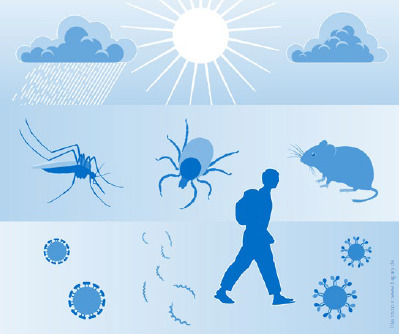
The climate (top) influences vectors and reservoir hosts as well as human behaviour (middle) and thereby vector- and rodent-borne pathogens (bottom) and the infectious diseases they cause Illustration: Guido Hegasy

This article provides an overview of these factors as well as an outlook on possible developments regarding different vector- and rodent-borne diseases that could be influenced by climate change (Figure 1). The first part of the article focuses on mosquito-borne viruses, which have regained importance in Germany as a consequence of globalisation and climate change. The next section considers tick-borne pathogens and the diseases they cause, including tick-borne encephalitis (TBE) and Lyme disease, which have high public health relevance for Germany. In the subsequent section, the zoonotic hantavirus disease in humans is described.

The final section lists recommendations for dealing with vector- and rodent-borne infections as well as for their control and prevention.

## 2. Methods

The authors jointly determined the content to be presented in this review according to their expertise. Relevant literature was identified according to the thematic aspects. In addition, reporting data collected at the Robert Koch Institute (RKI, Germany’s national public health institute) in accordance with the German Protection against Infection Act (Infektionsschutzgesetz, IfSG) were included to illustrate the situation in Germany. Individual groups of authors prepared drafts for the various sections, which were discussed and finalised by all authors.

## 3. Infectious diseases associated with mosquitoes

### 3.1 Occurrence and distribution of vector-competent mosquitoes in Germany

Mosquitoes can be vectors of viruses, protozoa and filariae. Until a few decades ago, they were known in Germany only as vectors of plasmodia (parasitic protozoa), the causative agents of malaria. After the widespread eradication of malaria from Europe in the mid-20^th^ century, mosquitoes did not play a role as vectors that needed to be monitored in Germany for a long time. The successful control of malaria was largely based on the development of synthetic drugs and the newly recognised insecticidal activity of DDT (dichlorodiphenyltrichloroethane) [1]. Mosquito-borne diseases characterised by relevant morbidity or even mortality were subsequently absent for decades. While the endemic *Plasmodium* species disappeared thanks to malaria control measures, the populations of the transmitting *Anopheles* species recovered and are still part of the present German mosquito fauna. This is composed of at least 52 species, with floodwater mosquitoes (*Aedes* (*Ae.*) *vexans, Ae. sticticus*) and the species of the *Culex* (*Cx.*) *pipiens* complex (*Cx. pipiens, Cx. torrentium*) being the most common and widespread mosquito species in Germany [2]. Five of these species are neozoa, i. e. species newly established in an area due to human influence, whose occurrence has been observed since 2007 as a consequence of globalisation and global warming. Continuous reproduction and repeated overwintering have been demonstrated for these species in Germany in the recent past, so that they must be regarded as established, at least regionally.

Since 2011, monitoring activities have been carried out continuously in Germany to provide up-to-date data on the occurrence and distribution of mosquito species. These are based both on the systematic recording of adults using trap catches and undirected larval collection (active monitoring), and on random submissions of mosquitoes from the public via the citizen science project ‘Mückenatlas’ (Mosquito Atlas, passive monitoring). In particular, the Mosquito Atlas has proven to be a good early warning system for detecting invasive species [3, 4].

For 23 of the species occurring in Germany, there is evidence that they are vector-competent for various pathogens, i. e. genetically and physiologically capable, in principle, of reproducing the pathogens in their bodies or bringing them to further development and transmitting them. This is also assumed to be the case for other native mosquito species, although reliable scientific evidence is still lacking [4].

The actual vector role of a blood-feeding arthropod is reflected by its vector capacity, which is, among other things, a function of vector frequency and the availability of infection sources [5]. Infection sources can be any vertebrates through which mosquitoes can become infected. The external temperature also plays a significant role in vector capacity, influencing both the development of the vector and of the pathogen inside the vector. At certain species-specific minimum temperatures, physiological processes are set in motion that accelerate with higher temperatures – up to maximum threshold temperatures. For example, after winter dormancy, some mosquito species continue larval development in spring at only a few degrees above freezing temperature (e. g. at 4 to 5°C for the Asian bush mosquito, *Ae. japonicus* [6]). With rising temperatures, there is an increase in biting frequency, acceleration of blood digestion, egg production, juvenile development, and generation cycle, resulting in higher overall population densities. Similarly, seasonal mosquito activity is extended. Counteracting this is a shorter individual lifespan at high temperatures [7].

Most arboviruses, i. e. viruses transmitted by arthropods, replicate and disseminate in their vectors only above temperatures of 11 to 15°C [8]. Within the tolerable temperature range of vectors and viruses, virogenesis runs faster the higher the temperature, so that the intensity and efficiency of pathogen transmission increases with higher mosquito biting frequency [9]. The fact that virus transmission by mosquitoes so far has been uncommon in Germany is possibly due – apart from the presumably moderate availability of infection sources – to the fact that temperatures have not been sufficiently high for the development of viruses in the vector and their efficient transmission. This is supported by laboratory experiments in which infected mosquito species in temperate climates became infectious at incubation temperatures of 24 to 27°C [10–12]. The first appearance of West Nile virus (WNV) in 2018, the warmest year to date since weather records began in Germany [13], also supports this hypothesis. Rising temperatures could thus enable the transmission of pathogens not only by known potential vectors, but also by native mosquito species not yet recognised as potential vectors.

Potential vectors of WNV, various *Culex* and *Aedes* species, occur throughout Germany [14]. The virus circulates seasonally between mosquitoes and birds, which carry the virus (transport hosts), develop a high viral load in the blood (viraemia), thus acting as amplification hosts, and sometimes die of the infection [15]. Based on previous outbreaks in southern Europe, cases of WNV infection had therefore been expected in Germany long before 2018 [16, 17]. Apparently, only the particularly high temperatures in late summer 2018 enabled efficient virus development in mosquitoes and visible transmission in the form of disease cases.

The main vectors of WNV to mammals, transmitting the virus from birds to other host groups (bridge vectors), are certain variants of the common house mosquito *Cx. pipiens*, especially late summer hybrids of the two biotypes *pipiens* and *molestus*, which do not discriminate between avian and mammalian hosts [18]. However, mammals themselves are not sources of WNV infection for mosquitoes (dead-end hosts) because the viral load in their blood is too low for viruses to be passed to mosquitoes. Since 2019, WNV has been detected several times in mosquitoes of the *Cx. pipiens* complex in Germany during the mosquito season and also in hibernating females of this species complex in the winter of 2020/2021 [19]. This proves the overwintering of the virus in the vectors and suggests its longterm persistence in Germany.

Due to evolutionary adaptation, endemic mosquito species cope well with the central European climate and can survive cold winters without any problems. Presence of infection sources, sufficient precipitation and availability of water reservoirs for larval development, species that have not yet appeared as vectors could also become relevant in the medium term if climate change and associated warming continues. Availability of water reservoirs is likely, especially in residential areas, due to the storage of rainwater in artificial containers for garden irrigation. In the long term, it must be assumed that mosquito species closely adapted to temperate conditions will shift their ranges to cooler regions and be replaced by species that prefer a warm climate.

Among the five new mosquito species that have become established in Germany since 2007, three are thermophilic (*Ae. albopictus, Anopheles petragnani, Culiseta longiareolata*) and three species are considered potential vectors of human pathogens (*Ae. albopictus, Ae. japonicus, Ae. koreicus*) [20]. As a result of poor climatic adaptation, thermophilic species find it difficult to establish and spread. Geographically, they occur only locally in Germany. However, their presence indicates improving living conditions for thermophilic species. Among these, the Asian tiger mosquito *Ae. albopictus* has a prominent position: it is considered the most invasive mosquito species worldwide [21] and is a highly efficient vector of numerous human pathogens [22, 23] (Section 3.3 Pathogens transmitted by *Ae. albopictus*). In southern Europe, where this mosquito is widespread and regionally occurs at high densities, it has been observed as a vector of dengue virus (DENV) and chikungunya virus (CHIKV) on several occasions [24].

The intercontinental spread of the tiger mosquito to Europe is mainly facilitated by the used tyre trade; its entry into Germany, however, is probably mainly due to long-distance motor vehicle traffic from southern Europe [25, 26]. Several populations have been established in Germany [27]. These can be traced back to strains that have adapted to climatic conditions and – in contrast to tropical strains – produce diapausing eggs, i. e. eggs characterised by a physiological resting phase enabling them to overwinter. Populations are predominantly found in the relatively warm Upper Rhine Valley and other areas of Baden-Württemberg, with Hesse, Bavaria and Thuringia also being affected [28].

In most places with known tiger mosquito populations, prevention and control measures have been implemented by (local) authorities (e. g. [29]). The primary goal of these measures is no longer the elimination of populations, but the reduction of population densities, as these determine the risk of pathogen transmission. Since mosquitoes have not been a health problem in central Europe in recent decades, expertise in vector management is currently very limited in Germany.

With increasing temperatures, the Asian tiger mosquito should find it easier to settle and spread in Germany. According to the literature, the limiting factor for settlement is the average annual temperature, which must be at least 11°C [30, 31]. Cold winters also cause problems for this mosquito; however, enough eggs usually survive in sheltered places and manage to establish a new population in the following year [32, 33]. According to the latest models, most areas in Germany will be suitable for *Ae. albopictus* colonisation by the year 2040, although different modelling approaches (e. g. mechanistic models, correlative niche models) lead to different outcomes. Lack of knowledge of climatic requirements and ecological adaptability of the tiger mosquito allow only rough predictions [34, 35]. The strongest limiting factors for the spread of *Ae. albopictus* in Europe are low winter minimum temperatures in the east, low summer average temperatures in the centre, and low precipitation in the south [36].

*Ae. japonicus* and *Ae. koreicus*, which have been shown to be able to transmit some viruses and nematodes in experimental studies [37–39], have also regionally established in Germany. They have not yet appeared as vectors in the field. Table 1 shows important mosquito species occurring in Germany that can act as vectors.

### 3.2 Pathogens transmitted by native mosquitoes

Some mosquito-borne viruses circulating in Germany, such as Sindbis, Batai, or Usutu viruses, are only mildly pathogenic or epidemiologically negligible, but the recently emerging WNV is of relevant public health importance. It belongs to the family *Flaviviridae* and was first isolated from a febrile patient in Uganda in 1937. Since the 1950s, it has been detected in mosquitoes, birds, and humans in many European countries [16]. An outbreak in Romania in 1996 was the first time it was associated with severe human illness and death in Europe [40]. In the last twenty years, increased epidemics have occurred on almost all continents [41].

During the hot summer of 2018, Europe experienced the largest recorded WNV outbreak to date, with more than 1,600 cases, including 166 deaths [42]. In contrast to previous large epidemics caused by WNV lineage 1 [16], WNV lineage 2 has been primarily circulating in Europe since 2010. Despite intensive and repeated investigations on the presence of WNV in German mosquitoes, birds, and horses, the virus was not detected in Germany until 2018 [13]. The same year saw the first reported human infection acquired in Germany (autochthonous infection); however, this infection was probably caused by direct contact with a dead bird. Human infection with WNV, as with all arboviruses, has been notifiable according to IfSG since 2016. In addition, there are overlapping notification requirements according to the German Transfusion Act (Transfusionsgesetz, TFG) [43]. Until 2017, all WNV infections reported in Germany were travel-associated. From 2019 to 2021, a total of 31 autochthonous human WNV infections were recorded in four German states (Berlin, Brandenburg, Saxony-Anhalt, and Saxony), with all of them presumably associated with mosquito bites (Table 2). All persons affected resided in regions where WNV infections in birds or horses had previously been described. Of these, 29 were symptomatic and thus fulfilled the reference definition of the RKI. The respective onset of disease was between July 27 and September 19, i. e. in or shortly after the hottest phases of the summer in the region. Ten women and 21 men aged 24 to 85 years were affected. Twelve infected persons had neuroinvasive infections, and of these, one patient died.

An estimated 80% of all WNV infections in humans are asymptomatic and about 19% develop West Nile fever without complications. About 1% of infected individuals develop a neuroinvasive form of the disease associated with a lethality of about 10%, predominantly the elderly and chronically ill [44]. The virus is also of great importance for blood safety [45], as individuals requiring transfusions are more susceptible to severe WNV disease due to their underlying conditions.

Only parts of Germany are currently affected by autochthonous WNV transmission, mainly Berlin, large parts of Brandenburg, Saxony and Saxony-Anhalt, as well as smaller parts of Thuringia and Lower Saxony [43]. This area, defined by virus detections in humans, birds, mosquitoes, or horses, has hardly changed since 2018 but may expand at any time. The infection is only seasonally relevant, in Germany mainly in the period from July to September.

The exact routes by which WNV reached Germany are not known; however, closely related virus strains circulate in Austria and the Czech Republic [13]. It is possible that the very intense 2018 WNV season in southern Europe provided increased opportunities for expansion of the endemic area. Good conditions for WNV circulation between mosquitoes and birds due to the exceptionally long and hot summer of 2018 probably allowed the first overwintering to occur in Germany [13].

Hotter summers, changing precipitation patterns, and people's recreational behaviour may influence the epidemiology of WNV in Germany: observed seasonality in Germany and data on outbreaks in other regions indicate that longer and hotter summers result in longer and more intense seasonal transmission of WNV. Precipitation patterns can influence populations of WNV-transmitting mosquitoes and thus contribute locally and regionally to the intensification of WNV transmission [46]. Last but not least, during particularly long and hot summers, people tend to spend more time outdoors and may come into contact with WNV-transmitting mosquitoes with a higher probability. A possible compensatory effect is that travel-associated WNV infections and other travel-associated arbovirus infections may become less frequent when summer vacations are spent in Germany instead of abroad due to warmer weather.

Preventive approaches are professional vector control, personal protective measures to reduce the risk of exposure (especially for older persons and people with pre-existing conditions), and screening of blood donations. Vaccines against WNV are currently not available for humans. Improved target group-oriented education is needed to raise people's awareness of WNV, allowing them to take personal measures.

### 3.3 Pathogens transmitted by *Ae. albopictus*

In addition to the yellow fever mosquito *Ae. aegypti*, the Asian tiger mosquito is the most important vector for arboviruses in the tropics and subtropics, including DENV, CHIKV and Zika virus (ZIKV), which are diagnosed quite frequently in travellers returning to Germany.

DENV infection leads to clinical symptoms in about 25% of cases. Most patients have a mild febrile and self-limiting illness known as dengue fever. A small proportion of patients develop a severe form of the disease, which may be associated with bleeding. Most ZIKV infections are asymptomatic. When symptoms do occur, they are usually mild and similar to those of a DENV infection. ZIKV infections during pregnancy, however, can result in foetal malformations (e. g. microcephaly). CHIKV infection often manifests as chikungunya fever. The main symptoms are severe muscle and joint pains, which can lead to a painfully hunched posture. In 5 to 10% of sufferers, joint pain can last for months.

The endemic areas of these viruses are located in the tropics and subtropics, including popular travel destinations such as Thailand, India and Brazil. Table 3 shows the number of infections with these pathogens reported annually to the RKI in Germany, in accordance with IfSG, over the last ten years. The case numbers fluctuate strongly, influenced by varying infection risks in the travel destinations. Except for one hospital-acquired (nosocomial) DENV infection and three ZIKV infections in the context of laboratory accidents, all infections were travel-associated.

Local *Ae. albopictus* can transmit viruses from symptomatic or even asymptomatic viraemic travellers returning to Germany to other people. This has led to CHIKV epidemics in Italy and small autochthonous case clusters of DENV and ZIKV infections in other southern European countries [47–49].

Mosquito-borne autochthonous infections with supposedly tropical viruses have not yet been documented in Germany. However, vector competence studies with German *Ae. albopictus* populations show that they are capable of transmitting CHIKV under prevailing summer temperatures [50]. In contrast, common central European summer temperatures are probably not sufficient for DENV and ZIKV epidemics [51, 52]. Warmer summers and prolonged hot spells are likely to promote autochthonous transmission of the aforementioned pathogens, as are years with an early, warm spring in combination with a summer that is not too dry, leading to high mosquito densities in mid-summer. The importance of an early, warm spring was highlighted by autochthonous dengue clusters in France in 2022 [53]. It should be noted, for example, that up to 75% of infections with DENV are asymptomatic and are therefore rarely diagnosed in Germany, while the infected individuals may still be relevant as a source of virus for mosquitoes. Reported infections with these pathogens must therefore be regarded as the tip of the iceberg. However, travellers who are no longer viraemic on arrival in Germany, who arrive in the cold season, or who have visited areas without *Ae. albopictus* (or other suitable vectors) do not represent a virus source for local mosquitoes.

## 4. Infectious diseases associated with hard ticks

### 4.1 Hard ticks as vectors in Germany and the influence of environmental and climatic factors

In central Europe, hard ticks are the most important vectors of infectious agents to humans. Among the at least 19 hard tick species native to Germany, which live in natural biotopes near suitable hosts, the castor bean tick *Ixodes (I.) ricinus* is most widespread throughout Germany and has the greatest significance for public health [54, 55]. This tick species is adapted to a very broad host range to ingest blood, finds particularly suitable conditions in oak-beech and mixed deciduous forests, in which it often occurs in high densities. All stages can feed on humans, although the juvenile stages in particular, with a size of about one millimetre, are easily overlooked. In addition, the meadow tick *Dermacentor (D.) reticulatus* and the ornate sheep tick *D. marginatus* are relatively widespread in some regions. The juvenile stages of these ticks usually parasitise rodents and can be detected in rodent burrows, their adults prefer larger mammalian hosts*.* Native hard ticks can transmit diverse pathogens such as TBE virus (Section 4.2 Tick-borne encephalitis), spirochaete bacteria of the genus *Borrelia* (Section 4.3 Lyme borreliosis), and other hitherto less noticed bacteria such as *Francisella tularensis (*causative agent of tularaemia), *Coxiella burnetii* (causative agent of Q fever), *Rickettsia* spp., *Anaplasma phagocytophilum, Ehrlichia* spp. and parasites such as *Babesia* spp. (e. g. causative agent of ‘canine malaria’ *Babesia canis*). Some of these pathogens cause more severe courses of disease and are therefore mostly notifiable in Germany according to IfSG or ordinances of the federal states. For others, cases of transmission to humans are recorded comparatively rarely, and infections in immunocompetent individuals are predominantly mild or unspecific, so that they often go unnoticed.

The infectious agents usually circulate among wild animals (reservoir hosts) and are transmitted among them mostly by hard ticks. Hard ticks are also considered to transmit these pathogens to other animal hosts such as livestock, domestic animals and humans. Prerequisites for transmission are exposure to infected ticks in their habitat, a sufficiently long attachment of the infected tick to the body for bloodsucking, and the absence of infection protection measures.

Further tick species and pathogens can also be introduced to Germany via wild animals, livestock, domestic animals or humans. For example, the repeated introduction via dogs of the brown dog tick *Rhipicephalus sanguineus* (vector of *Rickettsia (R.) conorii, R. massiliae*, and *Babesia vogeli*, among others) has been demonstrated since the 1970s, leading to its temporary establishment in buildings [56, 57]. Since the very warm years of 2018 and 2019, adult stages of the thermophilic species *Hyalomma (H.) marginatum* and *H. rufipes* have also been found in at least 12 of 16 German states [58–60]. They were probably introduced to Germany in their juvenile stages with migratory birds from Africa or southern Europe. Similar trends have been documented in several European countries, including Sweden, the United Kingdom, and the Netherlands [61–63]. *R. aeschlimanni*, a bacterium of the spotted fever group that is pathogenic to humans, was detected in about 30 to 40% of these ticks of the *H. marginatum* complex introduced to Germany. Crimean-Congo haemorrhagic fever virus (CCHFV) has not yet been detected in Germany [58, 59, 64]; however, *H. rufipes* infected with CCHFV was detected in a whinchat and a Western black-eared wheatear on the Italian island of Ventotene [65]. Increasing establishment of *H. marginatum* populations has been reported in southern Europe, Spain, and southern France, and isolated CCHFV detections in livestock, wild animals, and humans have been reported in southern Europe and Spain [66]. Whether previously non-native pathogens and tick species can establish in Germany and how the native host-tick-pathogen relationships already existing in Germany will develop depends on many factors, with climate change being a significant influence [67–69].

Ticks react directly to macro- and microclimatic factors in a species-specific manner, temperature and relative humidity being key factors. At higher temperatures and suitable humidity, for example, developmental processes are accelerated and thus the duration of moulting from one stage to the next or the duration of egg deposition is shortened [70–72]. Effects of climate change on native as well as medically relevant tick species with the potential of being introduced to Germany were investigated in more detail on behalf of the German Environment Agency [73, 74]. As a basis for modelling, georeferenced maps of Europe were produced for 17 tick species either native to Germany or with the potential for introduction, and the first tick atlas for Germany was published [75, 76] (current tick atlas published at [77]). *I. ricinus* is already widespread throughout Germany [74, 78] and in the course of climate change, higher tick abundances are possible in suitable biotopes, such as mixed oak-beech forests with undergrowth, sufficient hosts, and protective leaf litter. If, for example, under favourable climatic conditions, effective beech mast seeding occurs at shorter intervals, resulting in larger rodent populations [79, 80], more hosts are thus available for ticks such as *Ixodes* spp. and *Dermacentor* spp. to ingest blood and transmit pathogens. Temperature and humidity affect tick activity, developmental duration, diapause, overwintering, and survival rates. Individual active ticks have been observed at a soil temperature as low as 4°C [73].

Distribution modelling for *D. reticulatus* and *D. marginatus* using MAXENT and BIOCLIM models (statistical habitat models), taking into account biological and geographic features and using data about landscape and bodies of water, indicates that further distribution of these tick species, especially *D. reticulatus*, is already possible in Germany at present [74, 81]*.* This was confirmed by more recent detections of these tick species in other regions [82, 83]. Habitat suitability modelling was also conducted for *H. marginatum* using MAXENT and BIOCLIM models [74, 84]. A projection for 2050 indicates that climatic conditions for *H. marginatum* will continue to improve in Germany [74]. Detection of ticks of the *H. marginatum* complex in Germany since 2018 confirms the introduction and suitability of conditions for the development into adults for these ticks, at least in warm and drier spring and summer periods. Modelling, especially when also considering host populations, e. g. modelling the potential spread of *H. marginatum* by migratory birds [85], supports the risk assessment and indicates where to look for these ticks in the context of systematic monitoring for early detection. In general, however, there are still large uncertainties in such models due to the multifactorial nature of the events and the dependence on model assumptions.

Rising temperatures, especially in the winter to summer months, hot spells and extreme weather, as well as changes in the water balance affecting relative humidity and air saturation deficit, influence the natural foci of infections together with their plant and animal habitats, including the hard ticks and the pathogens they harbour. Further research is needed in order to better understand the complex relationships and apply this knowledge to prevention strategies. The two most significant diseases transmitted by hard ticks are addressed below.

### 4.2 Tick-borne encephalitis

In Germany, the TBE virus is transmitted primarily by *I. ricinus* ticks, less frequently by other tick species (including *D. reticulatus*) or via infected raw milk (products). About 70 to 95% of infections are asymptomatic. Symptomatic diseases are sometimes mild, sometimes more severe with manifestations of the central nervous system (CNS) like meningitis, encephalitis, and myelitis. Up to 1% of those with CNS symptoms die [86]. Older persons generally have more severe symptoms than children. Nevertheless, severe forms of the disease can also occur in children.

**Figure 2:**
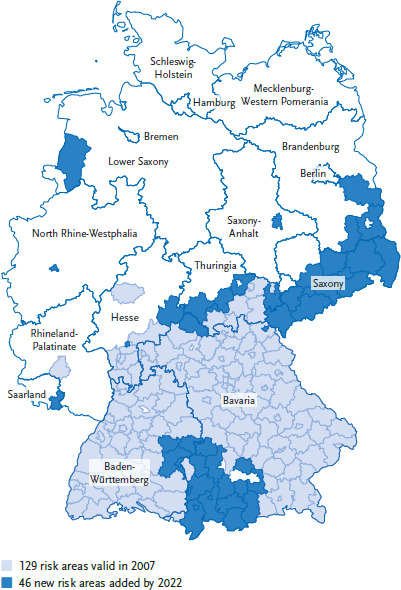
Development of tick-borne encephalitis risk areas, 2007-2022 Source: Robert Koch Institute

TBE cases reported in Germany since 2001 according to IfSG show a pronounced seasonality, with most cases (98%) occurring between April and November. Annual case numbers vary widely between 195 (2012) and 712 cases (2020). From 2001 to 2016, a median of 276 cases were reported annually. From 2017 to 2022, the median annual number of cases was 505, an increase of over 200 cases. A statistically significant trend of an annual 2% increase in case numbers was observed from 2001 to 2018, as well as a seasonal shift such that infections occurred 0.69 days earlier each year [87, 88]. While *I. ricinus* is widespread throughout Germany and can also transmit the causative agent of Lyme borreliosis over a wide area, the TBE virus occurs endemically mainly in the south of Germany, in the form of small-scale natural foci. The number of at-risk rural and urban districts according to the RKI definition (five-year incidence>1:100,000) increased from 129 districts in 2007 to 175 districts in 2022 [86], with a significant expansion toward the north (Figure 2). The development in Saxony is remarkable: in 2014, the first district was declared a risk area; by 2022, 10 of 13 counties were risk areas. Moreover, about 3% of the reported cases occur outside the official risk areas [86].

The TBE virus circulates in nature between its vectors (ticks) and its natural hosts (small mammals such as the bank vole *Clethrionomys glareolus* or the yellow-necked mouse *Apodemus flavicollis*). Virus occurrence is thus determined by a complex interplay of climatic and ecological factors, which can affect the transmission cycle in different ways. They may act synergistically on both biological systems (vector and host), or antagonistically on one partner in the transmission system at a time, or on both systems. This complex interaction complicates predictions or modelling of the future evolution of TBE.

Warmer temperatures, especially mild winters and warm springs, are beneficial for tick activity and survival. If the temperature is too high in hot and dry summers, the ticks retreat into protective vegetation layers [89]. Our own studies show that especially the number of nymphs (juvenile stage) is significantly increased in spring after mild winters, i. e. more tick larvae and/or nymphs survive. Overall, however, adult tick numbers do not appear to increase. Dry spells in subsequent months potentially result in increased mortality of adults or nymphs that do not develop into adults. For effects of climate change on rodent populations, such as the bank vole, findings are summarised below (Section 5 Hantaviruses). There are no analyses to date on direct effects on small mammals infected with TBE virus and thus indirectly on the transmission cycle of TBE virus. The influence of climatic factors on virus replication has also not been elucidated. However, there is evidence that certain TBE virus strains can adapt to different environmental temperatures within the tick [90]. An ecological niche model shows that the spread of TBE virus infection locations in Germany is significantly more frequent where precipitation and temperature are high in summer and few frost days occur in winter [91]. Given the foreseeable climatic changes, the habitat of *I. ricinus* may expand, especially in northern and eastern Europe, according to one modelling study [92]. Spread to higher altitudes has also been reported in the Czech Republic and elsewhere [93]. First TBE cases are regularly reported from countries previously considered TBE-free, e. g. the United Kingdom or the Netherlands in 2019 [94].

In addition to these abiotic factors, human behaviour also plays a role in infection risk. The record high of 712 TBE cases reported in 2020 was related not only to high tick occurrence but also to the fact that people went for more frequent walks during the COVID-19 pandemic [95]. Good conditions for mushroom picking can also lead to increased TBE virus infections in the fall. Warm periods can generally lead to increased time spent in nature and, if tick habitats are involved, to increased tick exposure and thus increased risk of infection.

Since there is no therapy to treat TBE, prevention is of great importance. Protective measures against tick bites, e. g. wearing long and light-coloured clothing, leaving no gaps between trousers and socks, and searching for attached ticks after each outdoor activity, can significantly reduce the risk of contracting the disease. In addition, TBE virus vaccination can effectively prevent infections [96]. In the majority (99%) of annual reported cases in Germany, people are unvaccinated or insufficiently vaccinated. Even in risk areas, only about 20% of the population has full vaccination protection [86]. This means that there is great potential for preventing the majority of TBE cases by increasing vaccination rates.

### 4.3 Lyme borreliosis

Lyme borreliosis (or Lyme disease) is by far the most common vector-borne infectious disease in Germany.

The bacteria *Borrelia (B.) burgdorferi* sensu lato (s.l.) transmitted by ticks (in central Europe mainly *I. ricinus)* are the causative agents of Lyme borreliosis, which can be accompanied by clinical manifestations of the skin (erythema migrans, acrodermatitis chronica athrophicans), nervous tissue (neuroborreliosis), joints (Lyme arthritis) and heart (Lyme carditis). Neuroborreliosis and Lyme carditis are sometimes associated with severe courses of disease. Lyme borreliosis occurs in all age groups and occurs, associated with the spread of its vectors, predominantly between the 40^th^ and 60^th^ parallel north, an area in which Germany and large parts of Europe are located.

Climate change may influence *B. burgdorferi* s.l. infections and the incidence of Lyme borreliosis within this area in a complex interplay of factors. It results in mild and wet winters and warmer springs in some regions. This extends the period of tick activity and density and increases the frequency of contact between humans and ticks, which leads to higher Lyme disease incidences. On the other hand, hot, dry summers are unsuitable for *I. ricinus* and may result in fewer infections. In addition, changing outdoor behaviours of people (e. g. spending time outdoors more frequently and earlier in the season) could alter the frequency of contact with ticks, leading to more frequent infections [97].

**Figure 3:**
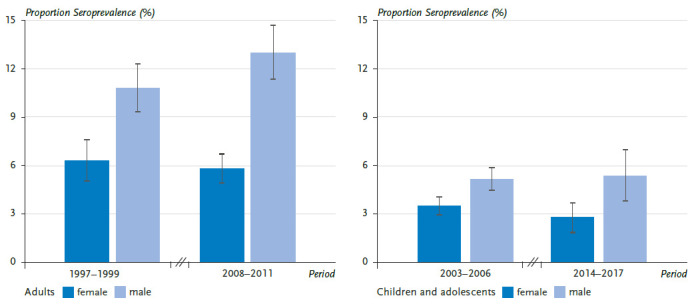
Representative estimates of *Borrelia burgdorferi* seroprevalence in adults (18-79 years) and children and adolescents (3-17 years) in Germany, 1997-2017 Source: Own representation after Woudenberg et al. [98].

In Germany, representative surveys of *B. burgdorferi* seroprevalence in children and adolescents as well as in adults are regularly conducted by the RKI and the National Reference Centre for *Borrelia* (Figure 3). During the periods observed, there was a slight increase in men (18–79 years) and a slight decrease in girls (3–17 years) [98, 99]. However, a significant increase in the seroprevalence of *B. burgdorferi*-specific antibodies in the overall population was not observed. Thus, a change in the risk of infection by 2017 could not be confirmed in the serosurveys so far. This could be due to the fact that influences with opposite effects (increasing temperatures and greater drought) have balanced each other out or had different regional effects, making them undetectable by nationwide surveys. Furthermore, the previous study periods of about ten years may have been too short to detect long-term trends.

Lyme borreliosis is a notifiable disease in 9 of 16 German federal states [100, 101]. The temporal occurrence of reported cases shows a distinct seasonal pattern. Onset of the disease between June and August was reported in 57% of cases, with seasonality varying by clinical manifestation: cases of erythema migrans peak in July (22%), neuroborreliosis cases in August (20%), and Lyme arthritis cases are more evenly distributed throughout the year. The incidence of Lyme borreliosis is higher in months with higher temperatures. This seasonal distribution varies slightly from year to year, but it has remained relatively constant across federal states to date. Relevant changes in the seasonal pattern cannot be confirmed so far. This could be because climate change has little or no effect on seasonal differences in Lyme disease incidence. Alternatively, the influence of climate change on seasonality could be undetectable in large-scale reporting data and effects may only be detectable on a smaller scale or be masked by artefacts in the reporting data.

Internationally, there are publications and reports from some regions that the distribution and incidence of Lyme borreliosis has increased in recent years in connection with warming (and thus more temperate winters and warm, humid summers), for example in the Midwest of the USA and in Canada [102]. Lyme borreliosis incidences have increased significantly in certain regions of Canada since surveillance began in 2009 [103]. This is primarily caused by a greater spread of the vector *I. scapularis*, mostly due to climate change. There has also been an increased spread of Lyme borreliosis in certain areas of northern Europe (e. g. Scotland) [104].

Modelling in the United States showed that in certain geographic areas (north-east), as temperatures continue to rise (according to climate scenarios), the incidence of Lyme borreliosis is likely to increase significantly in the coming decades [105]. However, these modelling efforts are subject to considerable uncertainty, and an increase in incidence could not be projected for all geographic regions considered. Another analysis in the United States found a significant association between temperature and incidence of Lyme borreliosis [106]. Assuming a 2°C increase in mean annual temperature over the next few decades, this study predicted an increase in cases of Lyme borreliosis of more than 20% in the United States.

A study from Austria evaluated the temporal and geographic trends of Lyme borreliosis and TBE for the period from 2005 to 2018 [107]. The incidences of the two diseases and their annual fluctuations were not geographically concordant, although the pathogens share the same tick vector and rodent reservoirs. Small-scale factors, some of which are still unknown, such as vector and pathogen biology and behavioural patterns, appear to play a role.

Although the factors influencing the incidence of Lyme borreliosis are complex, it can generally be assumed that climatic factors such as milder winters and warmer, more humid spring to autumn periods can lead to an increase in the incidence of infection and disease in certain smallscale regions.

## 5. Hantaviruses

**Figure 4:**
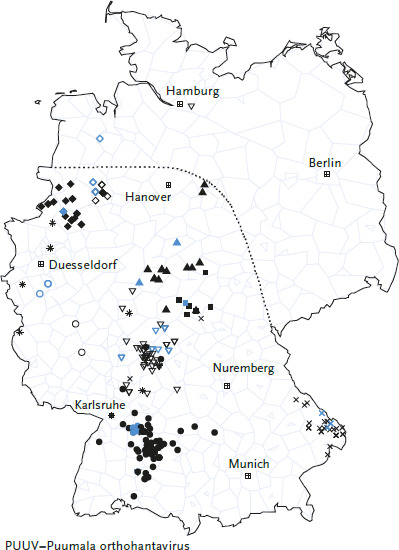
Distribution of Puumala orthohantavirus sequences in Germany. The symbols correspond to those in Figure 6 and show infections from known endemic areas in Germany. Multiple sequences from the same location are shown only once. Sequences originating from humans are depicted in black, those from bank voles in blue. The dotted line represents the hypothetical distribution boundary of PUUV-positive bank voles. Source: Own representation after Drewes et al. [108] and Weiss et al. [114 (CC BY 4.0)]

Rodents act as important reservoir hosts and vectors of various zoonotic pathogens: bacteria, viruses and protozoa, i.e. eukaryotic unicellular organisms. There are also a number of viruses that exist in rodents but are not thought to be transmitted to humans and are considered rodent-specific. In this section, hantaviruses will be discussed as the epidemiologically most important rodent-borne group of pathogens, since a significant increase in knowledge about the ecological background of the hantavirus disease has been recorded in recent years. At least nine different hantaviruses occur in Germany; most human disease cases are caused by the Puumala orthohantavirus (PUUV). Although the reservoir host of PUUV, the bank vole (*Clethrionomys glareolus*), occurs throughout Germany, PUUV infections have only been detected in bank voles in the southern, western, and north-western parts of Germany. Extensive studies have led to the postulation of a northern and eastern distribution boundary of PUUV in the bank vole (Figure 4) [108]. In contrast, Dobrava-Belgrade orthohantavirus (DOBV), genotype Kurkino, occurs exclusively in the eastern part of Germany. This distribution is caused by the occurrence of the reservoir, the striped field mouse (*Apodemus agrarius*), which is restricted to eastern Germany. Infections of the yellow-necked mouse (*A. flavicollis*), which occurs throughout Germany, were found only in the range of the striped field mouse. Although DOBV has also been molecularly detected in the yellow-necked mouse and a strain of DOBV has been isolated, it is unclear to what extent the yellow-necked mouse can transmit the virus to humans [109, 110]. Tula orthohantavirus (TULV) is found throughout Germany in the common vole (*Microtus arvalis*), but has rarely been associated with human infection and disease [111, 112]. Seoul orthohantavirus (SEOV) is endemic in rat populations in parts of Asia and has been repeatedly detected in domestic rats in Europe and the United States; in Germany, isolated infections have occurred in connection with foreign travel or after contact with pet rats [113]. In addition, five other hantaviruses have been described in Germany whose human pathogenicity has not yet been clarified.

Hantavirus disease in humans is a classical zoonosis. Infection occurs by inhalation of dust contaminated with saliva, faeces, or urine of infected animals, by contact of injured skin with contaminated materials, or rarely through bites. Infection through ingestion of contaminated food is also possible. A large proportion of hantavirus infections are asymptomatic or present with non-specific symptoms, so that diagnostic clarification is often not initiated and significant underreporting can be assumed. Symptomatic infections with the virus species that are relevant to humans in Germany (PUUV, DOBV Kurkino) usually present as a flu-like illness with fever, colicky, often unilateral flank pain, nausea and diarrhoea, headache and neck stiffness, visual disturbances (myopia, photophobia), and conjunctival haemorrhages [115]. In Germany, symptomatic disease mainly affects adults aged 20 to 60 years and is extremely rare in children. Men are significantly overrepresented in all age groups [116]. It is likely that age and sex significantly influence susceptibility to infection as well as disease severity. Exposure factors seem to play a minor role at most. Symptomatic hantavirus infections with laboratory evidence have been notifiable according to IfSG since 2001. Data of the reported cases are transmitted anonymously and electronically from the health offices to the respective state health office and from there to the RKI.

The epidemiology of hantavirus disease in humans is characterised by cyclic PUUV outbreaks occurring approximately every two to three years, mainly in the south, west and north-west of Germany [116]. DOBV Kurkino, which occurs in the north and east of Germany, only produces a small number of sporadic cases of disease and will not be discussed in detail in this article focusing on climate change.

**Figure 5:**
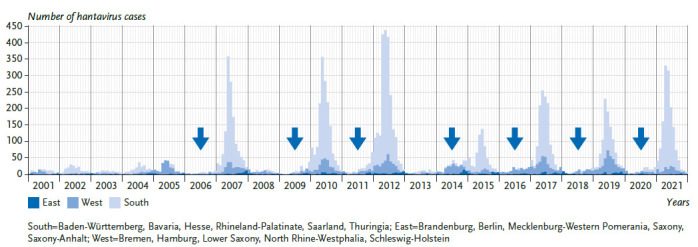
Reported hantavirus cases by region, year and month of disease onset in Germany, 2001-2021 (n=14.786 cases with known date). Arrows indicate beech mast years (>40% of beech trees showing medium or high fructification) in Baden-Württemberg [117]. Source: Own representation

**Figure 6:**
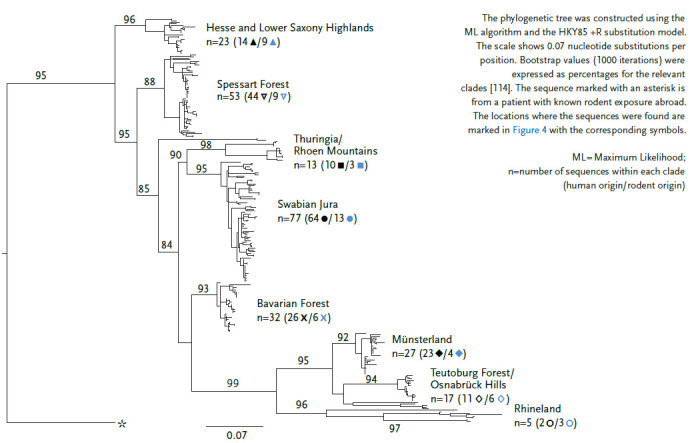
Phylogenetic analysis of Puumala orthohantavirus S-segment sequence based on a 504 nucleotide alignment of 258 sequences published in GenBank Source: Own representation after Drewes et al. [108] and Weiss et al. [114 (CC BY 4.0)]

With a mean annual incidence of 2.3:100,000 inhabitants in Germany, the six largest outbreak years, 2007, 2010, 2012, 2017, 2019, and 2021, contributed a large proportion of the total cases reported in Germany between 2001 and 2021 (n=11,464 of 15,823; 72.4%). In the remaining 15 years, the mean annual incidence was much lower at 0.35:100,000 inhabitants (Figure 5). The majority of cases (n=10,988 of 15,823; 69.4%) were reported in Bavaria and Baden-Württemberg, where known PUUV endemic areas are located (e. g. Swabian Alps, Lower Franconia, Bavarian Forest). The remaining federal states contribute relatively few cases, although endemic areas located there (e. g. the Osnabrück region or western Thuringia) also report locally high incidences in outbreak years. A detailed molecular analysis of PUUV outbreaks up to 2018, as well as current studies of bank voles at the postulated PUUV distribution boundary, show a separate evolutionary development of viruses specific to each endemic area over long periods of time (Figure 6). This suggests that the spatial extent of the different endemic areas is stable over time.

Hantavirus disease shows marked seasonality, with the case numbers peaking in spring or early summer (Figure 5). A gradual increase in case numbers through fall and winter seems to indicate an upcoming outbreak year. Nevertheless, hantavirus disease must also be considered outside this peak season; for example, a cluster of cases was reported in a small company in Lower Saxony in December 2017 [118].

The incidence of PUUV disease in the population is closely linked to the presence and abundance of bank voles [119]. A high population density of bank voles increases the transmission rate within the bank vole population, resulting in higher PUUV prevalence there [120, 121]. An increase in the population of infected reservoir animals increases the amount of virus-containing excreta in the environment and the likelihood that humans will come into contact with them. This, in turn, may lead to an increased rate of human infection. Therefore, when considering the influence of climate change on the future incidence of PUUV disease, it is critical to forecast future trends in bank vole populations. This in turn depends strongly on the future development of forests, especially beech forests.

The abundance of bank voles is subject to cyclic fluctuations. Mass reproductions are triggered by mast years, i. e. years in which there is above-average fructification of the bank voles’ food plants, mainly beech (*Fagus sylvatica*). Mass reproductions of bank voles regularly occur in years following mast years [119, 121].

The frequency with which mast years occur is climate-dependent. There is evidence that climate change has increased the frequency of mast years over the last hundred years [122, 123]. Consequently, the frequency of years with mass reproductions of bank voles has also increased, such that mast years followed by mass reproductions of bank voles now occur every two to three years (Figure 5). However, the increase in frequency of mast years is limited by the fact that a tree cannot produce a full mast in two consecutive years because above-average fruit production is associated with high physiological stress, which is evident through significantly reduced tree thickness growth in mast years [124]. However, it is possible that climate change could break the spatial synchronicity of mast events. This would result in mass fructification in beech trees occurring in a spatially heterogeneous manner [125, 126]. Such asynchronous mast events and their effect on bank vole populations have already been observed, suggesting that future outbreaks could be more localised rather than supraregional or nationwide [127]. Evidence also suggests that warm, wet winters favour transmission of PUUV within bank vole populations, regardless of their size and density, at least in northern Europe. Thus, climate may also have a direct influence on PUUV survival in the environment [128]. In Germany, however, climate change is more likely to affect forests by increasing drought. Based on observations of the drought years 2018 to 2020, it can be predicted that more than 30% of forested areas with beech as the main tree species and an important food source for bank voles are at risk [129]. In drought years, beech trees prematurely shed stunted beechnuts, despite having previously fruited well, providing only scarce food for bank voles [130]. Furthermore, it is likely that extreme weather events such as storms or heavy rainfall, in addition to forest fires as a consequence of drought, will have an impact on the occurrence of bank voles and PUUV. What consequences all these phenomena will have on bank vole populations and thus ultimately on the incidence of PUUV disease is not yet clear and must be the subject of further investigations. However, due to the above-mentioned natural limitation of the frequency of mast years as well as the expected negative climate effects on the growth and distribution of beech, a strong increase in the incidence of hantavirus disease in Germany in connection with the effects of climate change is not currently expected.

## 6. Recommendations

### 6.1 General recommendations

The following generally applicable recommendations can be made to protect the population from vector- and rodent-borne diseases in Germany:

(1) Strengthen science and research capacity on climate change and health to provide a more accurate assessment of the impact of climatic changes on vector- and rodent-borne infectious diseases

(2) Maintain or strengthen interagency One Healthbased collaboration in the health, environmental, and animal health sectors to ensure more effective interdisciplinary collaboration for infection prevention

(3) Expand monitoring of vector- and rodent-borne infectious agents in humans and animals

(4) Targeted information campaigns on infection risks and protective measures for the population

(5) Development of communication strategies for the medical profession

(6) Continuing education (a) of professionals related to behavioural prevention and health promotion in human and veterinary medical practices or facilities of the Public Health Service (Öffentlicher Gesundheitsdienst, ÖGD), (b) of employees in pest control companies, (c) of professionals working outdoors with an increased risk of infection, e. g. in forestry, and (d) of occupational health and safety specialists for these occupational groups

### 6.2 Targeted recommendations

The generally applicable recommendations are supplemented by the following targeted recommendations for Germany:

#### Infectious diseases associated with mosquitoes

(1) Promote the development of local response plans for WNV as well as the emergence of new vector-competent mosquito species (e. g. *Ae. albopictus*)

(2) Public information campaigns to prevent mosquito reproduction sites and the spread of new mosquito species

(3) Consideration of prevention measures, such as breeding site prevention or elimination, when planning of climate-resilient cities

(4) Sensitisation of the ÖGD to reported cases (Info box 1)

**Info box 1: Recommendations for the Public Health Service (ÖGD) with regard to DENV, CHIKV and ZIKV infections**

Local health authorities should seasonally point out the risk of further transmission in reported cases of suspected viraemic DENV, CHIKV and ZIKV infections in areas with *Ae. albopictus* and be alert for non-travel-associated secondary cases [131]. Physicians in relevant areas should also seasonally consider these pathogens, which are not endemic in Germany, in the event of clusters of illnesses with fever and/or skin rash and, if necessary, initiate appropriate diagnostics.

**Info box 2: Recommendations for the ÖGD with regard to tick-borne diseases**

Public health authorities should continue to provide preventive education about avoiding tick bites and removing ticks immediately. In addition, the public should also be informed about clinical manifestations, such as erythema migrans, in order to receive early medical attention and ensure early diagnosis and treatment, which can prevent more severe disease progression. A focus of these communication efforts could be on specific groups at increased risk of tick bites, e. g. people working in the forest or in public facilities in or near the forest (e. g. forest kindergartens, children’s and youth camps), members of Scouting associations, geocachers, people who collect mushrooms, or beekeepers.

#### Infectious diseases associated with hard ticks

(1) Assurance of correct tick identification as a basis for correct recognition of causal relationships

(2) Contact and exchange between scientists carrying out tick- and rodent-borne pathogen studies

(3) Development of effective and sustainable products, procedures, methods, and strategies to protect against tick infestation and pathogen transmission (including tick vaccine development, biological tick control, tick traps, effective repellents)

(4) Promote and conduct further representative surveys on the prevalence of *B. burgdorferi*-specific antibodies to detect a possible increase, which may be partly due to climatic factors

(5) Promote and conduct studies on the incidence and trends of erythema migrans in selected geographic regions

(6) Prevention measures and information by the ÖGD (Info box 2)

(7) Education of the public and the medical community about Lyme borreliosis (Info box 3)

#### Hantaviruses

(1) Increased dissemination of information on hantavirus infections to populations in affected areas, e. g. using the RKI fact sheet [132]

(2) Seasonal and targeted communication on the risk of hantavirus infections (Info box 4)

(3) Continuous surveillance of hantavirus infections in humans and monitoring of trends in the animal reservoir

(4) Networking of studies on various rodent- and vector-borne zoonotic pathogens

(5) Further development of hantavirus forecasting models and fine-resolution risk maps

(6) Promote and conduct studies on the synchronicity or increasing asynchronicity of bank vole mass reproductions

**Info box 3: Required communication strategies for Lyme borreliosis**

Misconceptions about the symptoms and diagnostic frequency of Lyme borreliosis exist in parts of the population, which have been spread for some time via social networks and blogging services. Individuals are sometimes incorrectly diagnosed with Lyme borreliosis and are subjected to prolonged suffering until they receive a correct diagnosis and therapy. Consequences include unnecessary burdens on the health care system, confused and dissatisfied patients, and the initiation of ineffective therapies such as antibiotic treatments. Communication strategies should educate the population about the disease, correct diagnostic options, and possible differential diagnoses of Lyme borreliosis.

**Info box 4: Communication on the risk of hantavirus infections to the population**

Education of the population on the risk of hantavirus infections and corresponding prevention measures should be carried out seasonally using selected communication strategies. In addition to direct information aimed at the general population, relevant community and interest groups should also be involved to provide targeted information to particularly exposed groups of people like forest workers and those working in pest control. While for PUUV in its distribution area predictions are possible, this is not yet the case for DOBV due to its sporadic occurrence. Therefore, general information should be provided in the distribution area of this virus (eastern part of Germany). The first detection of hantavirus infections caused by pet rats (Seoul orthohantavirus) has made clear that keepers and sellers of pet rats have to be informed about this risk; mandatory testing of pet rats for this and other pathogens would be desirable.

## 7. Conclusion

Higher temperatures, changing precipitation patterns, and human behaviour may influence the epidemiology of vector-and rodent-borne infectious diseases in Germany. The effects of climatic changes on the spread of vector- and rodent-borne infectious diseases need to be further studied in detail and considered in climate adaptation measures.

## Key statement

- International tourism and global trade in animals and goods contribute to the spread of vectors and pathogens.
- Factors like increasing temperatures, changing precipitation patterns, and human behaviour may influence the incidence and prevalence of vector- and rodent-borne infectious diseases.
- Biotic and abiotic factors influence vector activity, reproduction, development, overwintering, and survival.
- An ecological niche model showed that the distribution of locations with reported tick-borne encephalitis virus infection in Germany was associated with increased precipitation/temperature in summer and a decrease in frost days in winter.
- Prevention, especially vector control and exposure prevention, is of great importance for reducing the risk of infection with vector-and rodent-borne pathogens.
- The potential impact of climate change on human disease caused by Puumala orthohantavirus has not been conclusively determined.
- Further research is needed to better understand the impact of climate change on zoonotic pathogens, their reservoirs and vectors.

## Figures and Tables

**Table 1:** Important potential mosquito vectors in Germany and their epidemiological significance for the transmission of selected viruses

	WNV	DENV	CHIKV	ZIKV	USUV	SINV
*Aedes albopictus* (Asian tiger mosquito)	+	+++	+++	+++	?	+
*Aedes japonicus* (Asian bush mosquito)	+	+	+	+	+	?
*Aedes koreicus* (Korean bush mosquito)	?	?	+	+	?	?
*Aedes vexans*	+	?	?	+	?	?
*Culex pipiens* (common house mosquito)	+++	–	–	–	+++	+++
*Culex modestus*	++	?	?	–	?	?
*Culex torrentium*	+	?	?	–	++	+

+++ = high, ++ = medium, + = low, – = no, ? = unknown epidemiological significance

WNV=West Nile virus, DENV=dengue virus, CHIKV=chikungunya virus, ZIKV=Zika virus, USUV=Usutu virus, SINV=Sindbis virus

**Table 2:** Notifications of human West Nile virus infections since autochthonous emergence of the virus in Germany, 2018–2021 Source: SurvNet, database of infectious diseases with mandatory notification requirement in Germany

	Type of infections	2018	2019	2020	2021	Total
Notification according to IfSG	Travel-associated cases^1^	10	7	1	1	19
	Autochthonous cases^1^	1	5	20	4	30
	Autochthonous asymptomatic infections	0	0	2	0	2
Notification according to TFG only	Autochthonous infections in blood donors.	Not recorded	Not recorded	8	1	9
	**Total**	**11**	**12**	**31**	**6**	**60**

^1^ Symptomatic infections

IfSG=German Protection against Infection Act, TFG=German Transfusion Act

**Table 3:** Notifications of infections with dengue, chikungunya and Zika virus in Germany according to the German Protection against Infection Act, 2012–2021 Source: SurvNet, database of infectious diseases with mandatory notification requirement in Germany

	2012	2013	2014	2015	2016	2017	2018	2019	2020^2^	2021^2^
DENV	616	878	625	725	957	635	612	1176	205	60
CHIKV	9	16	162	110	74	33	26	88	26	4
ZIKV	0^1^	0^1^	0^1^	2^1^	22	69	18	11	6	2

^1^ No IfSG reporting obligation yet

^2^ Pandemic-related decline in long-distance travel activity

DENV=dengue virus, CHIKV=chikungunya virus, ZIKV=Zika virus, IfSG=German Protection against Infection Act
